# Design of the Prototype of Contact Drawing Device for Potential Individual Therapeutic Fiber Formation Purposes

**DOI:** 10.3390/pharmaceutics13060875

**Published:** 2021-06-13

**Authors:** István Sebe, István Kovács, Romána Zelkó

**Affiliations:** 1 University Pharmacy Department of Pharmacy Administration, Semmelweis University, Hőgyes Endre Street 7-9, 1092 Budapest, Hungary; sebe.istvan@pharma.semmelweis-univ.hu; 2 Cadminton Hungary Ltd., Mihály Munkácsy Street 46, 2120 Dunakeszi, Hungary; ikovacs@cadminton.com

**Keywords:** contact drawing, fiber formation, wound dressing, pharmaceutical use, microfiber, individual therapeutic need

## Abstract

Pharmaceutical compounding enables the preparation of unlicensed medicine to meet specific patient needs that do not have a licensed medicine available on the market. It must be performed in the best possible circumstance by certified pharmacists using validated standard operating procedures to obtain the highest quality medicinal product. The various spinning techniques provide drug delivery systems easily adapted to individual patient’s needs among the emerging technologies. The primary purpose of the present work was to introduce the prototype of a contact drawing device for the compounding of drug delivery systems for individual in-patient needs. The preliminary experiments resulted in oriented fibers of micrometer diameter range. The device can be placed in controlled conditions and could provide drug-loaded fibrous sheets for further treatments assuring the individual patient’s medicine need of the required quality.

## 1. Introduction

The development of tailored dosage provides a wide range of possibilities for meeting individual drug therapy needs. Hospital pharmacists may compound medications for their own patients if commercial products are unsuitable or not available or if therapeutic substitution is not feasible [1]. Custom-made medical devices (e.g., special wound healing scaffolds, patches, films) can also be prepared individually under pharmacy circumstances.

Techniques such as electrospinning (ES) can be used for the preparation of medical devices or drug delivery systems adapted to each patient’s requirements. The advantageous physical properties (high porosity, relatively small pore size) of electrospun meshes serve a dual purpose of allowing the wound to dry and keep out microbial contamination. The recently introduced portable electrospinning setup enables nanofibers to be applied at the bedside, bringing this technology closer to end-users treated in inpatient units. A portable setup over off-the-shelf electrospun nanofiber mesh has a few advantages, like the control of coverage area by nanofiber, nanofiber thickness control, and an that adhesive was not necessary to secure the nanofiber on the site [2,3].

Although, there are a few limits to the use of portable handheld devices. The device’s safety needs to be addressed before it can be put to use. At the same time, most electrospun mesh for wound dressing is based on solvent-based polymers, which cannot be considered inert from the point of their direct application on the skin. To avoid the solvent exposition, biocompatible and water-soluble polymer can be used, which does not readily dissolve when it comes into contact with water or fluid.

One of the commonly applied biocompatible polymers is polyvinyl alcohol (PVA), which is soluble in water at an elevated temperature but less so at a lower temperature. PVA may also be reinforced with additives such as cellulose nanocrystals to improve its mechanical properties and improve its high humidity stability [4].

In contrast to the limits of the application of portable devices in individual therapies, the contact drawing can provide a promising alternative by its simple use in controlled conditions using flexible changing of the composition in a relatively short operating time. In the drawing process, suspended fibers are fabricated by contacting a previously deposited polymer solution droplet with a sharp probe tip and drawing it as a liquid fiber, which is then solidified by rapid evaporation of the solvent due to the high surface area to volume ratio at reduced length scales [5].

An appropriate quality assurance system and a model procedure for risk assessment should be performed in hospital pharmacy circumstances in the course of patient-centred compounding, which is also highlighted in the EU Resolution CM/Res(2016)1 on the quality and safety assurance requirements for medicinal products prepared in pharmacies for the special needs of patients.

The authors propose a contact drawing method for fabricating polymer fibers continuously, in which polymeric micro-/nanofibers are formed by drawing and solidifying a viscous liquid polymer solution located in the reservoir of the device. By controlling the drawing parameters (pin diameter, temperature, revolution number), this method enables the formation of oriented networks of fibers having uniform diameters from micrometers down to upper nanometer-scales for different types and molecular weights of dissolved polymers.

The primary purpose of this study was to introduce a contact drawing device as a potential alternative for providing individual therapy in hospital pharmacy circumstances of inpatient units.

## 2. Materials and Methods

### 2.1. Materials for Fiber Formation

The fiber forming excipient was PVP 30 (polyvinylpyrrolidone, Kollidon 30, BASF, Ludwigshafen, Germany, approximate molecular weights: 50,000, glass-transition temperature values: 168 °C [6]), distilled water was selected as a solvent of the PVP 30 precursor solution.

### 2.2. Materials for the Contact Drawing Device

The frame construction of the device was made of steel (distr.: Metalloglobus Ltd., Budapest, Hungary). The sample holder containing the fiber-forming polymer solution has a total volume of 10 mL, made of acid-resistant/stainless steel (Tensile Strength: 520–720 MPa, distr.: Tímár Ltd., Budapest, Hungary). The spinning needles of the device are made of highly flexible acid-resistant steel (Prym, Stolberg, Germany). The chain drive for torque transmission is stainless steel (IWIS-Jwis Ltd., Germany/distr.: Power Belt Ltd., Budapest, Hungary), while the material of the V-belt is a fiber-reinforced plastic rubber (PIX-X’set^®^ 8 Section, distr.: S.I.S. Ltd., Budapest, Hungary). The frame of the collector collecting the fibrous web of the simple design is made of acid-resistant steel (stainless steel, Tensile Strength: 520–720 MPa, distr. Tímár Ltd., Budapest, Hungary), while the electrostatic product is made of thermosetting textile bakelite (distr.: Quattroplast Ltd., Budapest, Hungary). The collector plate of the simple design is made of Polytetrafluoroethylene (Teflon^®^, distr.: Quattroplast Ltd., Budapest, Hungary, yield stress: 22 MPa, melting temp.: 339 °C), while the electrostatic version is made of nickel-coated steel (distr.: Tímár Ltd., Budapest, Hungary) with a thin Teflon^®^ layer on the surface. The moving elements of the equipment were made by machining, and the rigid frame was made by welding.

### 2.3. Development of Contact Drawing Device

#### 2.3.1. Construction Development and Computer-Aided Design Modelling of Fiber Pad Weaver

The mechanical construction of the machine was built in CATIA V5 R18 SP4 (Service Pack) HF76 (Hotfix). Inbuilt components were created in the Part Design module through parametric 3D body modelling or were carried over from catalogue as standard components which are available on the market (like ball bearings, washers, studs and bolts or other fasteners). The entire construction was built together in the Assembly Design module. The alignment of each component was verified by adjusting all of the end positions which can be reached during the complex movement of the main arm, bed and frame. Synchronization of the components to each other happened during this phase. The technical documentation including necessary technical drawings for a patent application was created in the Drafting module. Views contain all information required for production and checking the quality of components subsequently. Bill of material (BOM) is listed for facilitating the production and acquisition of standard components. Finally, an animation was created for the complete dynamic verification in order to support the understanding of operation for the public audience.

#### 2.3.2. Contact Drawing Technique

**Figure 1:**
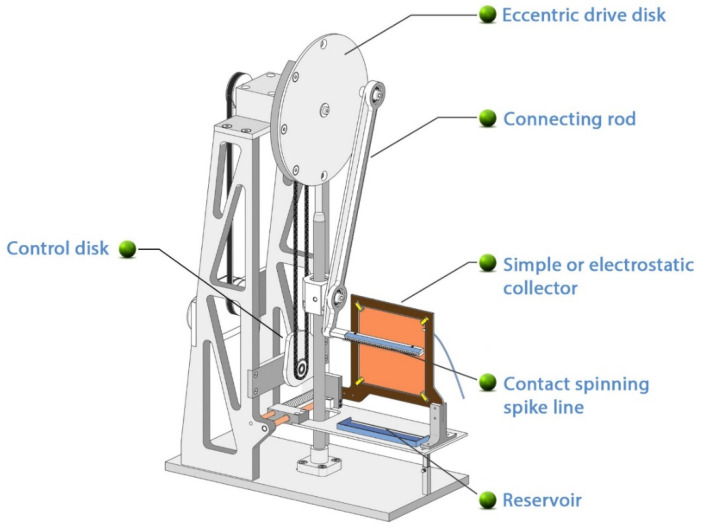
Three-dimensional view of contact drawing device.

**Figure 2:**
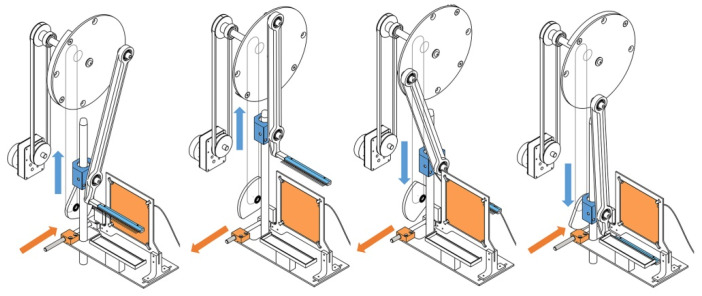
Motion steps of fiber formation.

Contact fiber formation is based on the principle that the contact fiber drawing device (Figure 1) can form fibers from liquid-phase, viscous polymer solutions through the cyclic motion methodology and form the obtained fibers into a sheet using a collector operating in synchronism with motion (Figure 2) [6].

**Figure 3:**
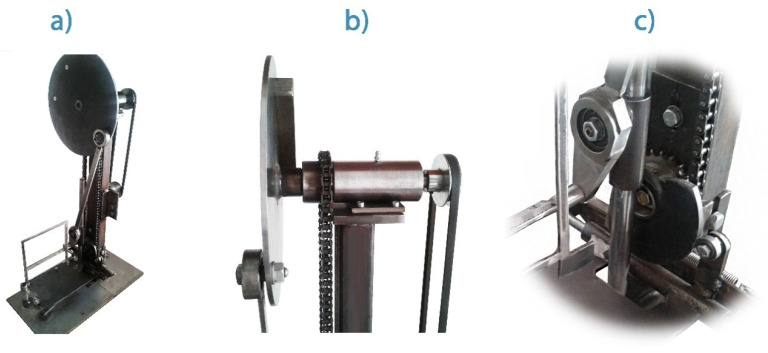
Photo about contact drawing device in full view (**a**), eccentric drive disc (**b**), guiding column, slider and control disc (**c**).

When operating the invention, the comb containing the thread pulling pins and the thread picking frame is moved. Mechanical motion is provided by the 9IDGE-120FP DKM220VAC (DKM Motor Co., Ltd., Incheon, Korea) type motor. The rotation of the 265 mm diameter eccentric disc at 120 revolutions per minute (RPM) is converted to linear motion. The prototype of the device and the main details responsible for its operation are shown in Figure 3. The reservoir containing the polymer precursor solution has a volume of 10 mL. A series of threading pins periodically immersed in the solution contains 39 pins with an effective length of 30 mm, a 1 mm upper-, and 0.2 mm contact diameter. The rotating speed was controlled with a toroidal transformer and measured with a laser revolution counter (DT-10L, Voltcraft, Hirschau, Germany). The collector has a base area of 142 × 142 mm. The contact drawing device has its own design with unique construction [7]. The productivity of fiber formation is 20 g/h on average.

#### 2.3.3. Sample Preparation

An aqueous viscous solution as polymer precursor solution was prepared with PVP K30 of pharmaceutical purity at 25 ± 1.0 °C temperature. The polymer powder was added slowly and in small portions to the distilled water with vigorous stirring to ensure that it disperses and dissolves rapidly without forming lumps. A gradual increase of PVP 30 was applied to determine the optimum range of weight percent of polymer concentration. The authors also investigated the extreme values of PVP 30 weight percent necessary for the spinning. The values below the optimum correspond to the weight percent of PVP 30 solutions, where fiber first formed, while above the optimum can be considered wherein fiber no longer created. The samples for this study were prepared based on this experience. The optimum concentration of the applied PVP and formulation parameters are listed in Table 1.

#### 2.3.4. Rheological Characterization of Polymer Viscous Solutions

Kinexus Pro rheometer (Malvern Instruments Ltd., Malvern, UK) was applied for the rheological measurements. Measured data were registered with rSpace for Kinexus Pro 1.3 software (Malvern Instruments Ltd., Malvern, UK). The PVP 30 precursor solutions were measured using a cone and plate geometry. The gap between the cone and plate of sample placement was 0.15 mm. The temperature of the samples was controlled by Peltier system of the rheometer with an accuracy of ±0.1 °C. Viscometric properties were determined at 25 °C for an hour with a shear rate of 1.0 s^−1^.

#### 2.3.5. Scanning Electron Microscopy Imaging of Contact-Spun Fiber Sample

For tracking the fiber morphology, Scanning Electron Microscopy (SEM) (JEOL 6380LVa, Tokyo, Japan) was applied after gold coating of the fibers. Samples were fixed by conductive carbon adhesive tape. The measuring parameters were the following: accelerating high voltage: 20 kV; working distance: 12 mm. The average diameters of the fibers were determined based on the diameters of 50 different randomly selected individual filaments [8].

## 3. Results and Discussion

### Development of Contact Drawing Device

Figure 2 and Figure 3 show a prototype of the contact drawing device, indicating the main components. During operation, each component is subjected to a complex movement in which microfibers are formed by contact of the spike/needles line and the solution. It performs a cyclic movement through the slider and the comb that fits on the driving rod. The slider moves in a straight line, while the comb moves in an elliptical path.

A stage in a plane parallel to the base plate secures the collector and the 10 cm^3^ reservoir containing the polymer solution, and moves them horizontally at the same time. During the entire period of movement of the comb, it moves from one end position of the stage to another and then returns to its initial position. The movement is coordinated so that it reaches the highest position of the comb along one side of the collector and its lowest position along the opposite side. In the lowest position, the fiber spikes are immersed in the viscous polymer solution in the reservoir and, in moving to the uppermost position, draw thin fibers, thus forming a thin, tissue-like layer on a collector rotatable in its plane by 90°. The rotation of the control disc forces the stage to move through the control buck and roller. The return of the stage to its initial position is ensured by a spring.

The fiber spinning device itself ensures the production of sheets, made up of a solution phase of thin fibers, containing the fibers parallel or perpendicular to each other. A metal plate aids the fibers’ orientation and fixation during formation, and the collector is placed inside the support frame. The collector can be energized in a more complex design. The electrostatically charged plate attracts the fibers and fixes them in position, thus helping to orient the fibers formed in further cycles relative to each other. The mechanically stable final shape of the insert is created by rotating the fiber picking frame 90° in its plane at least once during a production period. Rotation results in the formation of transverse fibers.

**Figure 4:**
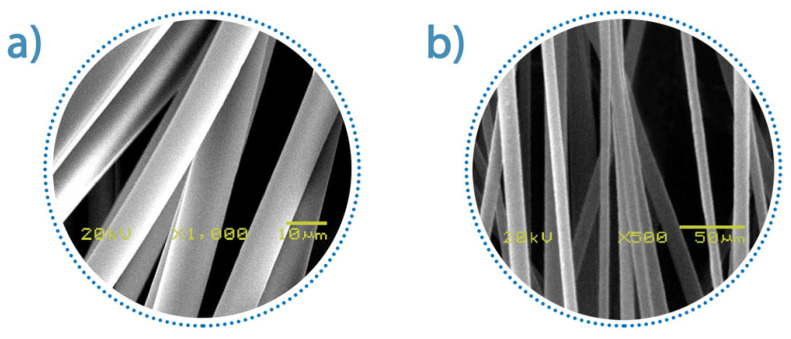
SEM image of contact-spun PVP 30 fiber (**a**), Magnification: 1000×, (**b**) Magnification: 500×.

All polymers with physicochemical properties suitable for rotary-spun microfibers can form contact fibers as well [9]. Of the pharmaceutical polymer excipients, the PVP 30 should be highlighted [10]. The prototype was also tested with PVP 30 polymer. Figure 4 illustrates the aligned fibers formed from the viscous PVP30 solutions. At lower concentrations, only a few inhomogeneous fibers with beads can be obtained. When increasing the polymer concentration, the stretching force generated by the mechanical motion was not enough to draw fibers of uniform diameter. Experience has shown that fibers with uniform morphology and thickness can be formed from a precursor solution by contact movement. The rate of cyclic motion must reach a minimum value for a given polymer, which was 120 RPM in the experiment. It can be assumed that by increasing the applicable speed above the minimum limit, the fibers’ average thickness can be reduced.

## 4. Conclusions

By constructing and testing a prototype of the contact fiber spinning equipment, it was proved that polymer microfibers with uniform morphology, and a microfiber web composed of fibers, could be created through mechanical movement and contact methodology. All of this potentially makes the process suitable for producing therapeutically useful drug-loaded delivery systems from drug-containing polymer precursor solutions that can be used directly for microfiber sheets.

The advantage of this process is that it offers a new, simple preparation method for the formation of fibers for pharmaceutical and medical use, which can even be carried out in clinical pharmacy conditions. The device creates an oriented fibrous structure from separable individual parallel and quasi-perpendicular intersecting fibers, formed into directly usable parallel sheets.

The fiber diameter can be varied in the same operating cycle using pins of different diameters; thus, a fibrous sheet of individual fibers of varying diameters can be obtained.

**Figure 5:**
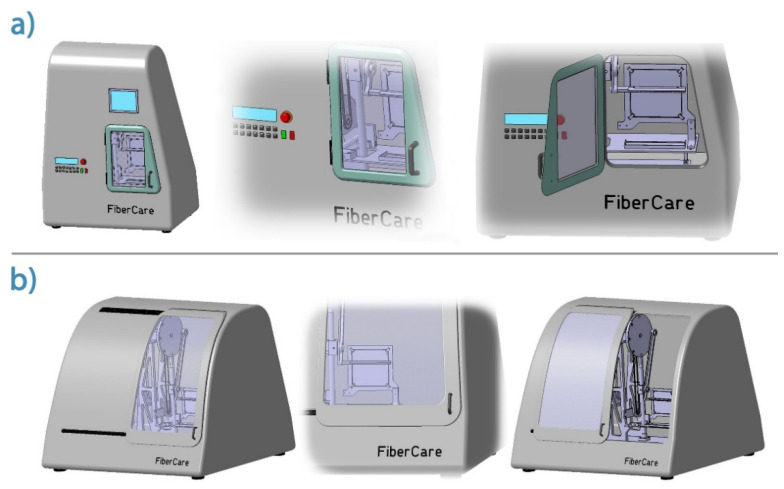
Three-dimensional views of various visual designs ((**a**) outer views with controlling elements, (**b**) inner views) of future ’ready for clinical use’ contact drawing device (FiberCare).

Figure 5 shows a visual design of a compact clinical embodiment of the device to be developed. The formulation conditions (temperature, humidity, period time) can be controlled and monitored by external control.

## Figures and Tables

**Table 1:** Formulation parameters and average diameter of contact-spun fibers.

Applied Polymer	PVP 30
Revolution per Minute (RPM)	120
Relative Centrifugal Force (RCF)	2
Concentration in Aqueous Medium (%*w*/*w*)	60–65
Viscosity of the PVP 30 precursor solutions (Pas)	28.0–28.5
Average Diameter of Fiber (μm)	8.2 ± 1.3

## Data Availability

Not applicable.

## References

[1] Visser J.C., Wibier L., Mekhaeil M., Woerdenbag H.J., Taxis K. (2020). Orodispersible films as a personalized dosage form for nursing home residents, an exploratory study. Int. J. Clin. Pharm..

[2] Liu G.-S., Yan X., Yan F.-F., Chen F.-X., Hao L.-Y., Chen S.-J., Lou T., Ning X., Long Y.-Z. (2018). In Situ Electrospinning Iodine-Based Fibrous Meshes for Antibacterial Wound Dressing. Nanoscale Res. Lett..

[3] Mouthuy P.-A., Groszkowski L., Ye H. (2015). Performances of a portable electrospinning apparatus. Biotechnol. Lett..

[4] Peresin M.S., Habibi Y., Vesterinen A.-H., Rojas O., Pawlak J.J., Seppala J.V. (2010). Effect of Moisture on Electrospun Nanofiber Composites of Poly(vinyl alcohol) and Cellulose Nanocrystals. Biomacromolecules.

[5] Nain A.S., Wong J., Amon C.H., Sitti M. (2006). Drawing suspended polymer micro-/nanofibers using glass micropipettes. Appl. Phys. Lett..

[6] Prudic A., Kleetz T., Korf M., Ji Y., Sadowski G. (2014). Influence of Copolymer Composition on the Phase Behavior of Solid Dispersions. Mol. Pharm..

[7] Sebe I., Zelkó R., Kállai-Szabó B. (2015). Gyógyszerészeti és orvosi alkalmazású kontakt szálhúzó berendezés és eljárás polimer alapú, hatóanyag-tartalmú lapkák előállítására. Szabad. Közlöny Védjegyértesítő.

[8] Sebe I., Kállai-Szabó B., Kovács K.N., Szabadi E., Zelkó R. (2015). Micro- and macrostructural characterization of polyvinylpirrolidone rotary-spun fibers. Drug Dev. Ind. Pharm..

[9] Sebe I., Kállai-Szabó B., Oldal I., Zsidai L., Zelkó R. (2020). Development of laboratory-scale high-speed rotary devices for a potential pharmaceutical microfibre drug delivery platform. Int. J. Pharm..

[10] Rogalski J.J., Botto L., Bastiaansen C.W.M., Peijs T. (2020). A study of rheological limitations in rotary jet spinning of polymer nanofibers through modeling and experimentation. J. Appl. Polym. Sci..

